# Development and validation of a nomogram for predicting pelvic lymph node metastasis and prognosis in patients with cervical cancer

**DOI:** 10.3389/fonc.2022.952347

**Published:** 2022-09-02

**Authors:** Mengting Wang, Min Ma, Liju Yang, Chengtong Liang

**Affiliations:** ^1^ Department of Obstetrics and Gynecology, The Affiliated Hospital of Yangzhou University, Yangzhou University, Yangzhou, Jiangsu Province, China; ^2^ Department of Laboratory Medicine, Clinical Medical College, Yangzhou University, Yangzhou, Jiangsu Province, China

**Keywords:** cervical cancer, lymph node metastasis, SEER, nomogram, prognosis

## Abstract

**Objective:**

Cervical cancer (CC) is one of the main causes of death among gynecological malignancies. Patients with CC with lymph node metastasis (LNM) have poor prognoses. We investigated the risk factors and prognosis of LNM in patients with CC patients using data from the SEER database.

**Methods:**

We collected the information of cervical cancer patients registered in SEER database from 2010 to 2015. The dataset was divided into a training set and a validation set at a 7:3 ratio. LASSO regression analysis was used to evaluate risk factors for LNM in patients with CC. Using the results, we established a nomogram prediction model. C-index, ROC curves, calibration curves, decision curve analysis, and clinical impact curves were used to evaluate the prediction performance of the model.

**Results:**

We included 14,356 patients with CC in the analysis. Among these, 3997 patients were diagnosed with LNM. A training set (10,050 cases) and a validation set (4306 cases) were used for the following analysis. We established nomogram LNM prediction models for the patients with T_1-2_-stage CC. The C-indices for the internal and external validations of the prediction models were 0.758 and 0.744, respectively. In addition, we established a prognostic nomogram for all CC patients with LNM, and the internal and external validation C-indices were 0.763 and 0.737.

**Conclusion:**

We constructed a quantitative and visual predictive nomogram that predicted prognosis of patients with LNM in CC to provide clinicians with a reference for diagnosis and treatment.

## Introduction

Cervical cancer (CC) is one of the most common gynecological malignancies and is the fourth leading cause of cancer-related death in women. In 2018, there were 569,847 new cases of CC worldwide, with more than 300,000 deaths (1). In developing countries, the incidence of CC ranks second among female tumors. Approximately 85% of CC-related deaths occur in developing countries (2). However, CC is also a significant health risk in developed countries. The prognosis of patients with metastatic CC is poor (3). Metastasis of CC can be categorized as follows: direct spread, lymphatic metastasis, and hematogenous metastasis. Lymphatic metastasis is the major form of metastasis, and significantly impacts prognosis (4). Lymph node metastasis (LNM) can provide a reference point to guide postoperative adjuvant treatment of CC (5). The FIGO clinical staging system was revised in 2018 to include reassessment of lymph node status and tumor size. According to FIGO staging (2018) (6), micrometastasis or macrometastasis to lymph nodes is classified as stage IIIC, regardless of size or parametrial invasion, and requires concurrent chemoradiotherapy. Isolated tumor cells do not alter the FIGO stage due to unclear clinical impact. Accurate assessment of lymph node metastases is crucial for developing individualized treatment regimens, improving prognosis, and reducing mortality (7).

New growth and morphological changes in lymphatic vessels (lymphangiogenesis) may be conducive to the entry of tumor cells into the lymphatic network, resulting in metastasis and diffusion. Furthermore, development of new blood vessels in new tissue growth areas is an important factor in tumor proliferation and diffusion. A study by Tantari et al. (8) showed that early cervical cancer did not require new lymphatic endothelial cell proliferation for lymphatic diffusion. This indicates the importance of pre-existing pericancerous lymphatic vessels in metastasis of early cervical cancer. Patients with early CC with pelvic LNM account for about 10% to 30% of all patients with CC (9, 10). FIGO reported that the 5-year survival of patients with stage IA-V CC with negative LNM was 94.1%, while patients with positive LNM had a survival rate of about 64.1% (11, 12). Diagnosis of LNM in patients with CC is associated with a significantly lower 5-year survival rate. In addition, accurate evaluation of LNM is dependent upon choice of surgical methods. Although pelvic lymphadenectomy may be improve long-term survival of patients with CC with LNM, extensive lymphadenectomy may lead unnecessary complications such as blood vessel/nerve damage, infection, lymphocyst formation, and lymphoedema of lower limb (13–15). Lymphadenectomy can seriously affect the quality of life of patients, prolong hospital stays, and endanger lives (16). Selective lymph node dissection may lead to the incomplete removal of metastatic lymph nodes, resulting in increased risk of recurrence and metastasis.

Evaluation of LNM in patients with CC is typically performed using imaging technology, and determination of metastasis is mainly based on lymph node size (17). However, preoperative examinations such as endoscopic ultrasonography and computed tomography are not accurate tools for evaluation of LNM (11, 18, 19). Therefore, clinicians must make a comprehensive judgments based on high-risk clinicopathological factors.

The sentinel lymph node (SLN) is the first site of tumor metastasis through lymphatic vessels, and has high reference value for disease diagnosis and treatment. Thus, sentinel lymph node biopsy (SLNB) has been increasingly accepted and recommended by clinicians as a diagnostic tool. Patients with early-stage CC who undergo SLNB experience reduced short-term postoperative lymph node-related complications, reduced sensory and motor impairment, and improved quality of life compared with patients who undergo SLNB + pelvic lymphadenectomy (PLND). Mathevet et al. randomly performed SLNB or SLNB+PLND in 206 patients with early-stage CC (20). Their results showed that lymphatic morbidity in patients who received sentinel node resection alone (SN arm; 31.4%) was significantly lower than that in patients who underwent SN+PLND (51.5%; P=0.0046). Postoperative neurological symptoms were also lower in the SN group than in the SN+PLND group (7.8% vs. 20.6%, P= 0.01, respectively). These results indicated that SLNB is a safe technique for patients with early-stage CC. Ancillary analysis from Favre et al. (21) showed that there was no significant difference in 4-year disease-free survival between the two groups. Wydra D et al. (22) conducted a study of SLN that included 100 patients with early CC, and found that the detection rates using the SLN were as follows: I_b1_ stage: 96.6%; I_b2_ stage: 66.7%; II_a_ stage: 62.5%; the false negative rate was 3%. False negative rates are relatively low when the tumor diameter is < 2 cm. Therefore, SLNB is considered reliable for diagnosis of early-stage CC. However, the diagnostic accuracy of SLNB is affected by tumor size, staging, and other factors. Overly large cervical tumors can compress the lymphatic vessels, resulting in poor drainage of the biopsy dye, and difficulty with diagnosis. Therefore, early evaluation of the SLN is critical to accurate diagnosis. Intraoperatively resected SLNs used to diagnose cervical cancer require routine frozen section analysis. However, several studies (10) have found that intraoperative frozen section assessment is less accurate for detection of micrometastases. Slama et al. (23) showed that frozen section analysis of SLNs from 225 patients with cervical cancer resulted in detection of micrometastases in only 2 of 17 patients. The overall sensitivity of frozen section analysis for all types of metastases was 56% mainly because the method was unable to detect lymph node micrometastases and was unable to detect isolated tumor cells.

Nomograms are commonly used in medicine to evaluate the occurrence of risk events. Nomograms can aid in development of integrated biological and clinical models to enable use of personalized medicine to help clinical decision-making (24). The suggested nomogram in our study may guide SLN biopsy: when nomograms suggest a high risk of lymph node metastasis, SLN mapping should be carefully done; In case of low risk of SLN metastasis, missing SLN raises the question of the benefit of pelvic lymphadenectomy according to mSKCC algorithm. Therefore, used a nomogram to establish an accurate, sensitive, cost-effective, and non-invasive tool for patients with CC with LNM that can help clinicians determine whether a patient at high risk, and to predict survival rate. This nomogram can be used as a reference for diagnosis and treatment.

## Materials and methods

### Data collection

The surveillance, epidemiology, and results database (SEER database) was established by the National Institutes of Health in 1973. It is one of the most representative large-scale cancer databases in North America. We obtained data for patients with CC registered in the SEER database (SEER*Stat Software version 8.3.5, https://seer.cancer.gov/data/) from 2010 to 2015. The site code ICD-O-3 (International Classification of Diseases for Oncology-3)/WHO 2008 was restricted to ‘cervix uteri.’ The exclusion criteria were as follows: 1) Age < 20 years; 2) diagnosed by autopsy or death certificate; 3) unknown survival time/metastasis status; 4) unknown LNM status. Finally, 14,356 patients diagnosed with CC were included in the analysis; Among these patients, 3997 were diagnosed with LNM. We stratified the patients into different cohorts according to age, race, grade, AJCC stage, T stage, M stage, histology, surgery, regional lymph node surgery, radiotherapy and chemotherapy, other distant metastasis sites (bone, brain, liver, and lung), tumor size (for the convenience of statistics, we choose 5cm/10cm as the dividing point), and marital status. Patients were divided into a training set (10,050 cases) and validation set (4306 cases) at a 7:3 ratio. The baseline characteristics of the data set are shown in **Table 1**. Chi-squared test was performed according to table 1.

**Table 1:** Baseline characteristics of patients with cervical cancer.

Variables	Training set	Validation set	χ^2^	*P*
	n = 10,050 (%)	n = 4,306 (%)	
**Age**			2.14	0.54
≤39	2503 (24.90)	1137 (26.40)		
40-59	4823 (47.99)	2005 (46.56)		
60-79	2345 (23.33)	988 (22.94)		
≥80	379 (3.77)	176 (4.08)		
**Race**			0.91	0.64
White	7495 (74.57)	3137 (72.85)		
Black	1450 (14.43)	650 (15.09)		
Others	1028 (10.23)	480 (11.14)		
**Grade**			0.65	0.89
I	853 (8.48)	374 (8.68)		
II	3202 (31.86)	1340 (31.19)		
III	3103 (30.97)	1298 (30.14)		
IV	123 (1.22)	83 (1.92)		
**Histology**			0.23	0.97
SCC	7721 (76.82)	3243 (75.31)		
AC	1261 (12.54)	546 (12.68)		
ASC	267 (2.65)	146 (3.39)		
NOS	801 (7.97)	371 (8.61)		
**AJCC**			1.43	0.70
I	4478 (44.56)	1889 (43.87)		
II	1423 (14.16)	644 (14.95)		
III	2461 (24.49)	1021 (23.71)		
IV	1553 (15.45)	692 (16.07)		
**T stage**			2.46	0.48
T_0-1_	5314 (52.87)	2233 (51.86)		
T2	2435 (24.22)	1000 (23.22)		
T3	1723 (17.14)	765 (17.76)		
T4	317 (3.15)	187 (4.34)		
**M stage**				
			0.07	0.94
M0	8822 (87.78)	3721 (86.41)		
M1	1228 (12.22)	585 (13.58)		
**Tumor size (cm)**			1.02	0.60
<5	4069 (40.48)	1687 (39.18)		
5–10	2610 (25.97)	1176 (27.31)		
>10	180 (1.79)	81 (1.88)		
**Surgery**			8.51	0.80
None	4733 (47.09)	2062 (47.88)		
Local tumor destruction/excision	1212 (12.06)	488 (11.33)		
Total hysterectomy (simple, pan-)	2263 (22.52)	932 (21.64)		
Radical/extended hysterectomy	1748 (17.39)	771 (17.90)		
NOS	94 (0.93)	53 (1.23)		
**The number of removed regional lymph nodes**			0.87	0.65
None	6590 (65.57)	2847 (66.12)		
1–3	221 (2.19)	82 (1.90)		
≥4	3116 (31.00)	1302 (30.23)		
**Chemotherapy**			0.65	0.42
No/Unknown	4718 (46.94)	2059 (47.81)		
Yes	5332 (53.05)	2247 (52.19)		
**Radiotherapy**			0.02	0.96
No/Unknown	4141 (41.20)	1811 (42.06)		
Yes	5909(58.79)	2495(57.94)		
**Bone metastasis**			4.44	0.35
No	9915 (98.66)	4185 (97.19)		
Yes	135 (1.34)	121 (2.81)		
**Brain metastasis**			0.99	0.75
No	10,012 (99.62)	4289 (99.61)		
Yes	38 (0.38)	17 (0.39)		
**Liver metastasis**			1.91	0.17
No	9856 (98.07)	4212 (41.91)		
Yes	194 (1.93)	94 (2.18)		
**Lung metastasis**			3.13	0.77
No	9739 (96.90)	4111 (95.47)		
Yes	311 (3.10)	195 (4.53)		
**Marital status**			1.64	0.65
Single	3156 (31.40)	1332 (30.93)		
Married	4051 (40.31)	1642 (38.13)		
Divorce/Separation	1417 (14.09)	642 (14.91)		
Widowed	895 (8.90)	400 (9.29)		

Number of variables with unknown status are not shown in the table above. Details are as follows: Race (77, training set; 39, validation set); Grade (2769, training set; 1211, validation set); AJCC (135, training set; 60, validation set); T stage (261, training set; 121, validation set); Tumor size (cm) (3191, training set;1362, validation set); Regional lymph node surgery (123, training set; 75, validation set); Marital status (531, training set; 290, validation set). NOS, Not Specified.

### Establishment and verification of nomograms

We used regularized regression (LASSO regression) to analyze influencing factors related to LNM of T_1-2_-stage CC and factors associated with prognosis of all CC patients with LNM. We established two nomogram prediction models. The C-index, ROC curve, calibration curve, decision curve analysis, and clinical impact curve were used to evaluate the stability and reliability of the models. Statistical analysis and picture generation were performed using R software (4.1.3).

## Results

### Baseline characteristics of the study population

**Figure 1:**
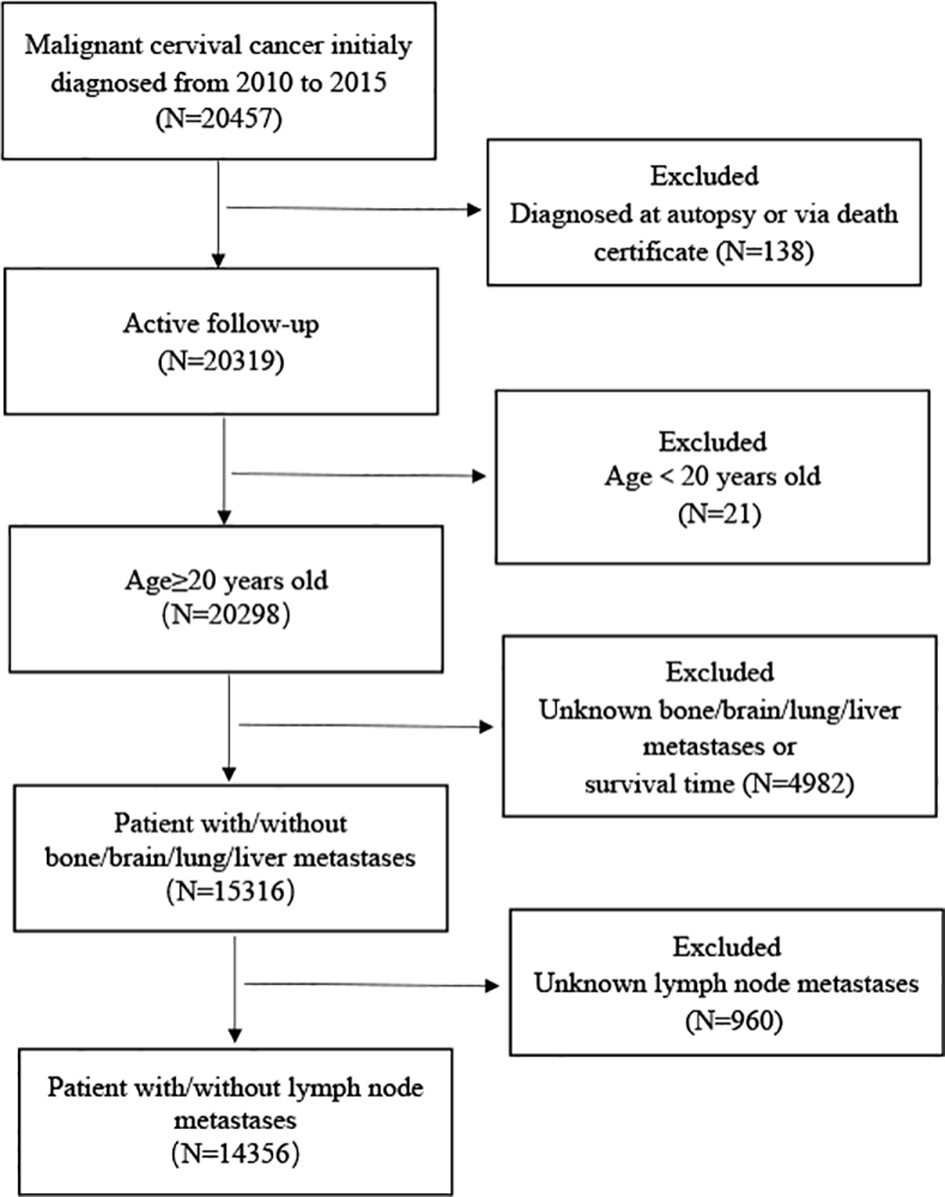
Flow chart showing the study design and patient selection.

From 2010 to 2015, 20,457 patients were diagnosed with CC, including 5413 patients with LNM (26.46%). In addition, 6101 were excluded, resulting in inclusion of 14,356 patients with CC in the analysis. Flow chart showing the study design and patient selection (**Figure 1**). 10,050 (70%) patients were placed in the training set and 4306 (30%) were placed in the validation set. There were 7721 patients with SCC (76.82%) in the training set and 3243 (75.31%) patients with SCC in the validation set. Patients with SCC accounted for the majority of all pathological types. Most patients were in the 40–59 age group (4823 cases, 47.99% in the training set, and 2005 cases, 46.56% in the validation set). Patients without distant metastasis accounted for the vast majority (8822 cases, 87.78% in the training set, and 3721 cases, 86.41% in the validation set). More patients had lung metastasis (311 cases, 3.10% in the training set, and 195 cases, 4.53% in the validation set) than bone, brain, or liver metastasis. The characteristics of the data set are shown in **Table 1**. There were no differences between the training and validation sets (*P*>0.05).

### Screening for risk factors

In the analysis of risk factors for LNM T_1~2_-stage CC, 12 factors (namely age, race, grade, histology, T stage, M stage, tumor size, marital status, lung metastasis, liver metastasis, brain metastasis, and bone metastasis) were evaluated to determine if they were associated with LNM. After statistical analysis, eight factors with nonzero coefficients (age, coefficients -0.129; race, coefficients -0.031; grade, coefficients 0.047; T stage, coefficients 1.134; M stage, coefficients 2.287; tumor size, coefficients -0.118; lung metastasis, coefficients -0.092; marital status, coefficients -0.046) were included in the nomogram prediction model of LNM in T_1-2_-stage CC. In addition to the 12 factors mentioned above, the effects of treatment methods (surgery, regional LN surgery, radiotherapy, chemotherapy) and AJCC stage on prognosis were analyzed during development of the prognostic model. Thirteen risk factors with nonzero coefficients (age, coefficients 0.138; race, coefficients 0.013; histology, coefficients 0.149; T stage, coefficients 0.220; M stage, coefficients 0.533; tumor size, coefficients 0.060; lung metastasis, coefficients 0.442; liver metastasis, coefficients 0.390; bone metastasis, coefficients 0.220; surgery, coefficients -0.210; regional LN surgery, coefficients -0.085; radiation coefficients -0.346; chemotherapy, coefficients -0.723) related to prognosis were identified, and were included in the nomogram.

### Evaluation of the established nomogram

**Figure 2:**
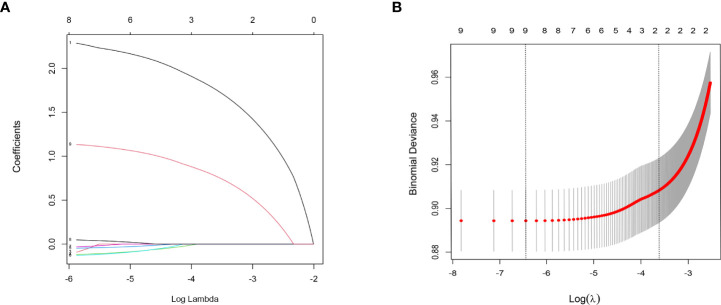
LASSO regression analysis was used to screen out factors associated with LNM. Analysis of T_1-2_-stage CC: **(A)** LASSO coefficients 1.M stage 2. Liver metastasis 3. Lung metastasis 4. Tumor size 5. Marital status 6. Age 7. Race 8. Grade 9. T stage; **(B)** Selection of the tuning parameter (λ).

**Figure 3:**
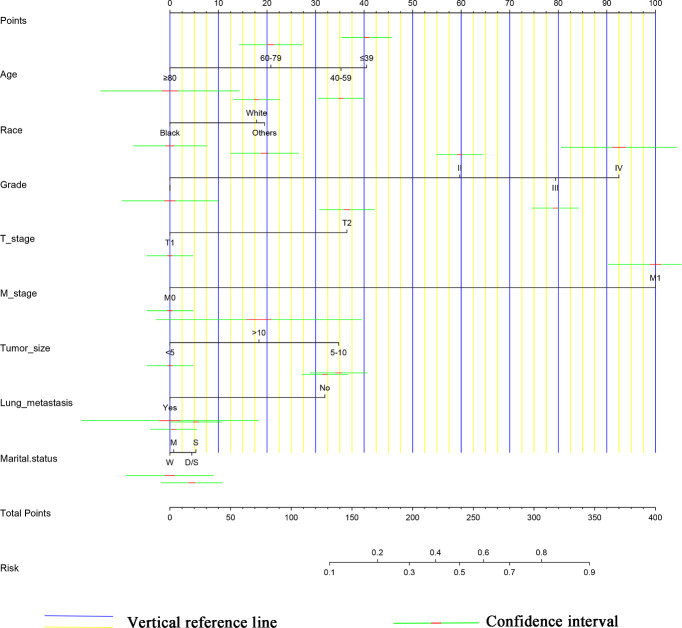
Nomogram for T_1-2_-stage CC. S, Single; M, Married; D/S, Divorce/Separation; W, Widowed.

**Figure 4:**
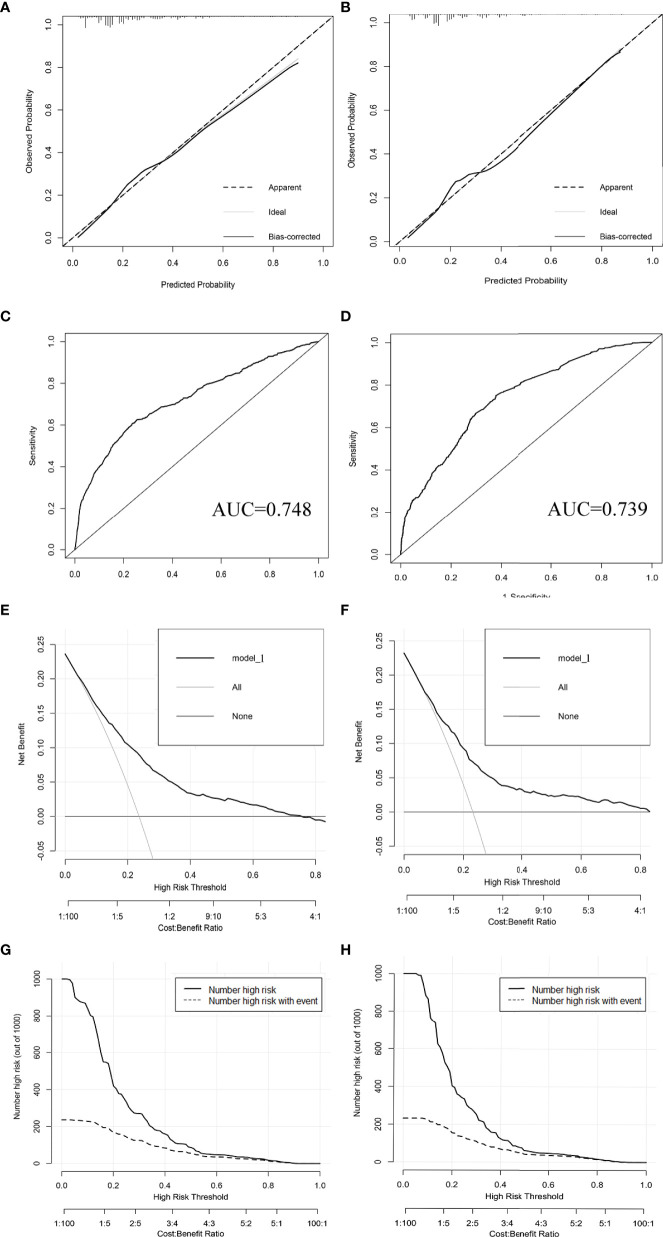
Calibration curves **(A, B)**, ROC curves **(C, D)**, decision curve analysis **(E, F)**, and clinical impact curves **(G, H)** were used to evaluate the prediction performance of the T_1-2_-stage CC nomogram. **(A, C, E, G)** for internal validation; **(B, D, F, H)** for external verification.

**Figure 5:**
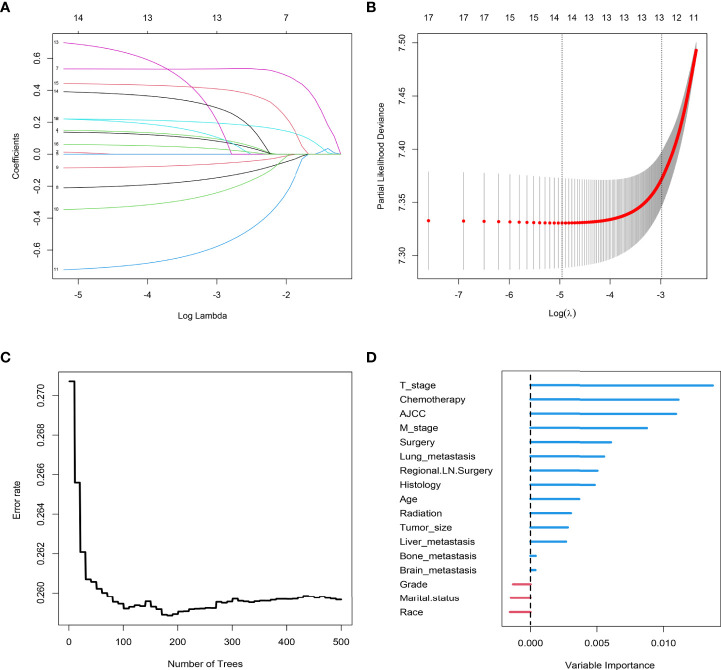
LASSO regression analysis was used to screen out factors related to the prognosis of patients with CC with LNM: **(A)** LASSO coefficients **(B)** Selection of the tuning parameter (λ). Random forest analysis, **(C)** forest tree and **(D)** Importance score.

**Figure 6:**
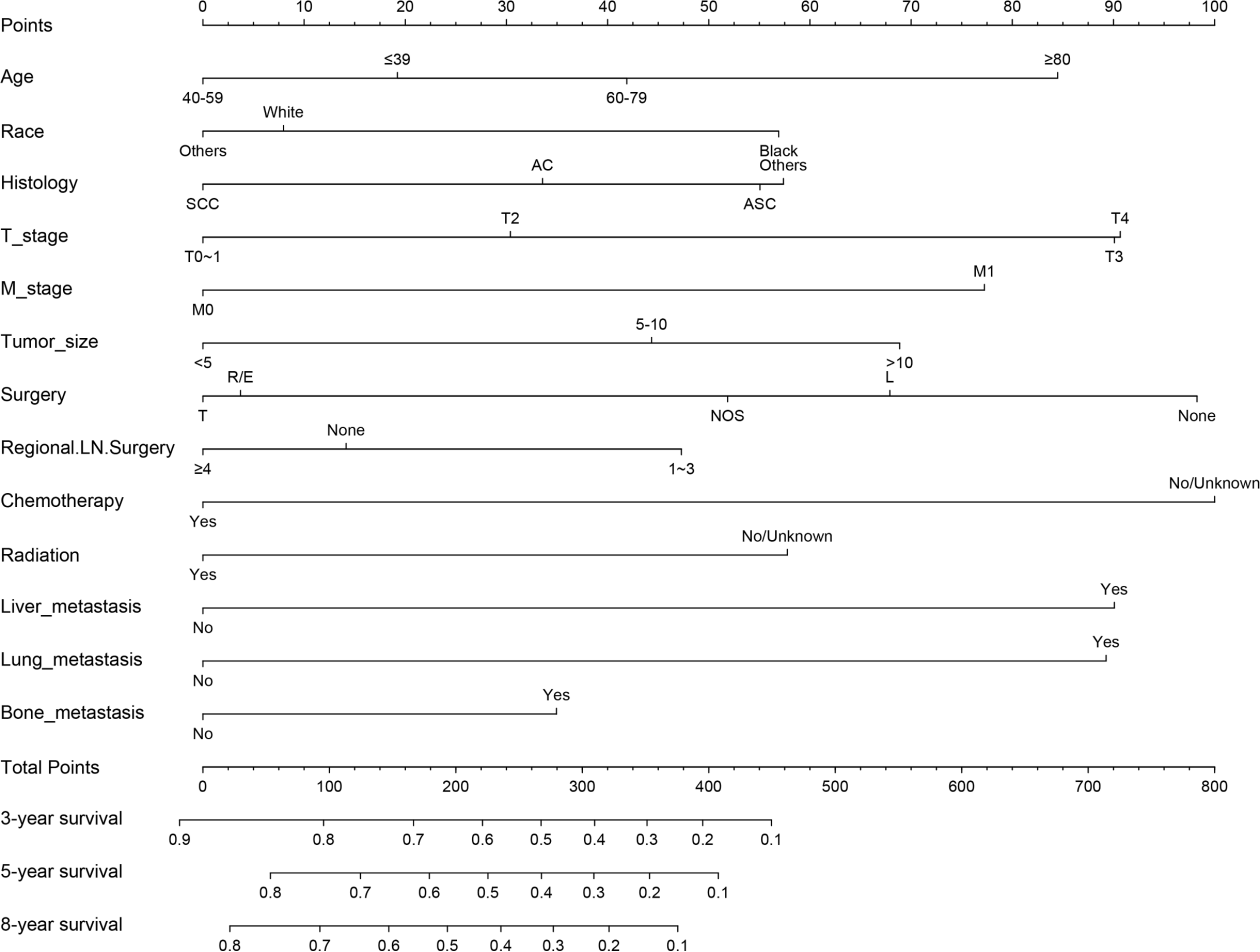
Nomogram for prognosis (overall survival) of patients with CC with LNM (Model 2). SCC, Squamous cell carcinoma; AC, Adenocarcinoma; ASC, Adenoskvamous carcinoma. L, Local tumor destruction/excision; T, Total hysterectomy (simple, pan-); R/E, Radical/extended hysterectomy.

**Figure 7:**
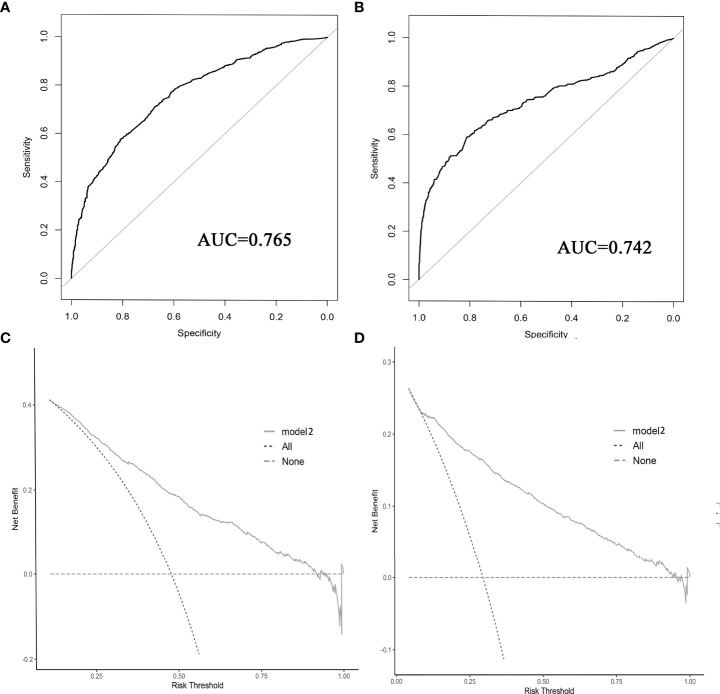
ROC curve **(A, B)** and decision curve analysis **(C, D)** were used to evaluate the prediction performance of the prognostic nomogram. **(A, C)** for internal validation; **(B, D)** for external verification.

**Figure 8:**
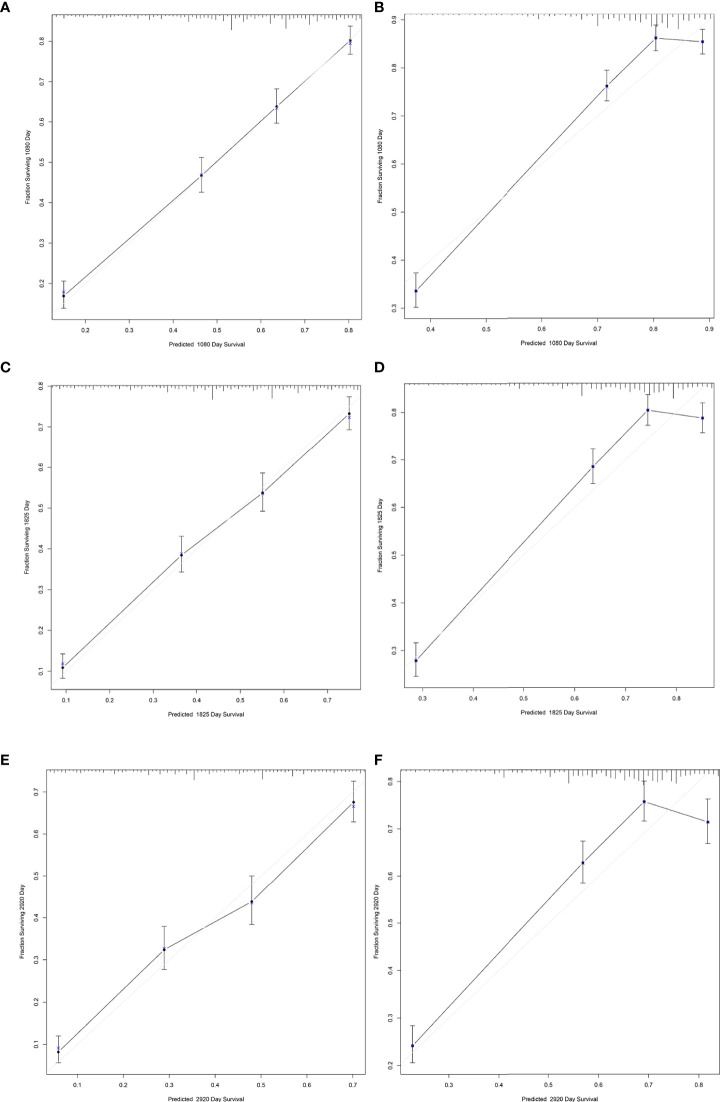
Calibration curves for predicting 3-, 5-, and 8-year survival of patients with CC with LNM. **(A, C, E)** for internal validation; **(B, D, F)** for external verification.

The results of LASSO regression analysis are shown in **Figure 2** for patients with CC with LNM. In the established nomogram prediction model (**Figure 3**) for LNM of T_1-2_ CC, the C-indices were: 0.758 (training set) and 0.744 (validation set). The areas under the curves (**Figure 4**) were: 0.748 (95% CI: 0.743–0.760, training set) and 0.739 (95% CI: 0.711–0.766, validation set). The calibration curve, decision curve analysis, and clinical impact curve are shown in **Figure 4**. LASSO regression and random forest analysis (**Figure 5**) were performed to identify factors associated with prognosis of patients with CC with LNM. In the established nomogram model (**Figure 6**) for prognosis of patients with CC with LNM, the C-indices were: 0.763 (training set) and 0.737 (validation set). The areas under the curves (**Figure 7**) were: 0.765 (95% CI: 0.742–0.783, training set) and 0.742 (95% CI: 0.716–0.757, validation set). The decision curve analysis is shown in **Figure 6**. The 3-, 5-, and 8-year correction curves are shown in **Figure 8**.

## Discussion

Risk factors for pelvic LNM in patients with CC has been a constant focus of clinical and scientific research, particularly with regard to prediction of LNM in early-stage CC. According to the 2018 edition of the National Comprehensive Cancer Network (NCCN) guidelines in the United States, the risk factor most strongly associated with early CC is LNM. Lymph node metastasis significantly impacts treatment and prognosis of CC (9, 10). Therefore, accurate evaluation of LNM aid in designing treatment regimens for patients with CC to improve their prognoses. To screen associated clinical risk factors, LASSO regression analysis was used in this study. The strength of LASSO regression is the ability to perform variable screening and complexity adjustment while fitting a generalized linear model, resulting in less error. All variables can be analyzed simultaneously to avoid overfitting and to reduce the impact of collinearity. The article of Yi et al. (25) only focused on the prognosis of cervical cancer patients with lymph node metastases. We had not only established and verified a nomogram for predicting the occurrence of LNM in patients with T_1-2_-stage CC, but also constructed and verified a nomogram for predicting survival of patients with CC with LNM. Among the pathological factors related to LNM of CC, maximum tumor diameter and tumor invasion have been consistently reported as independent risk factors. Studies have shown that the risk of LNM was higher when the tumor diameter was >2 cm (26). Horn et al. (27) and Turan et al (28) proposed that tumor diameter >4 cm significantly increased the rate of pelvic LNM. Another study also found a positive correlation between tumor diameter and risk of LNM (OR: 1.073–4.286 (29). These findings are consistent with the conclusion of our study that larger tumor diameter is associated with greater risk of LNM. Tumor diameter can reflect growth time of a tumor. Tumor growth is a process of continuous invasion and proliferation.

As tumor diameter increases with growth duration, the depth of invasion will increase. The contact area between the tumor and the lymphatic vessels increases over time, leading to increased risk of LNM. Cibula et al. (30) found that the depth of cervical invasion was associated with pelvic LNM. Depth of interstitial infiltration greater than 1/2 of the entire layer is a risk factor for LNM. Parauterine interstitial blood vessels and lymphatic vessels are abundant. As depth of invasion increases, the range expands. Cancer cells readily metastasize along lymph and blood vessels. Tumors extending to the vagina are also associated with pelvic LNM of CC. Tumors that extend to the vagina are typically larger and may grow actively. Tumors grow around, then invade, the lymphatic system. Studies have shown that pelvic LNM was 2.46 times higher in tumors involving the vagina than in those without vaginal involvement. A report from Matsuo et al. (31) showed that survival rates for stage IIIC1 varied significantly with T stage (5-year rates: 74.8% for T1, 58.7% for T2, and 39.3% for T3). The results obtained in our study are similar to those from other studies. Our nomogram showed that T stage was predictive of LNM, and T stage was an independent prognostic factor. We showed that younger individuals were at higher risk for LNM. This finding was consistent with the finding of a study by Kim (32). Higher risk for LNM may be related to a higher degree of malignancy in young patients (33). Young patients are more metabolically active than older patients, therefore tumors grow more rapidly, which may contribute to LNM and distant metastasis. In addition, LNM may not be sensitive to chemotherapy due to local necrosis and insufficient oxygen, which may affect prognosis. There is some disagreement with regard to histopathological grade and LNM of CC. Bai et al. (34) stated that grade was an independent risk factor for pelvic lymph nodes in CC. They indicated that low grade CC was highly malignancy and grew rapidly. Poorly differentiated tissues are more similar to interstitial tissues, so they are more prone to metastasis. Poorly differentiated tumors are less sensitive to chemoradiotherapy, resulting in poor prognosis. The results of our study confirmed that poorly differentiated and undifferentiated tissues were more prone to LNM. Recently, the incidence of non-squamous histologic CC has increased. Special types of CC have low incidence but have biological characteristics such as strong invasiveness, short course of disease, rapid progression, early metastasis, and poor prognosis. We confirmed this in our prognostic analysis. A previous report indicated that 60% of patients with squamous cell carcinoma do not have pelvic LNM (35), and non-squamous histology is an independent risk factor for LNM (36). In contrast, our study did not include pathological types in the prediction model of LNM.

The clinical impact of LNM is dependent on the types of metastases, the localization of nodes, and the number of involved nodes. According to the new 2018 FIGO classification, patients with micrometastases (MIC) or macrometastases (>2 mm) are classified as IIIC1 in the case of pelvic involvement or IIIC2 in the case of paraaortic involvement, while the presence of isolated tumor cells (ITC) does not impact disease classification. However, the clinical impact and treatment of low-volume lymph node metastases remain controversial. A meta-analysis by Guani et al. (37) concluded that MIC had a negative effect on DFS and OS, while ITC was not significantly associated with DFS or OS. These results indicated that MIC should be considered true metastasis and treated accordingly. Para-aortic metastases are associated with poorer prognosis. Para-aortic lymph node metastasis is the most common distant metastasis of cervical cancer. Once para-aortic lymph node metastasis occurs, the 5-year survival rate of cervical cancer patients drops to 20% to 40% (38). As for the clinical impact of the number of LNM (In more than 50% cases, only one node is involved), the current consensus is that the more the number of lymph nodes metastasis, the worse the prognosis of patients.

T stage, histological type, and tumor grade are intrinsic characteristics of tumors and have been shown to be independent prognostic factors for patients with CC and patients with CC with LNM. According to the clinical practice guidelines of the International Federation of Gynecology and Obstetrics, the standard surgical scheme for early CC (I A2–II A2) is radical hysterectomy + pelvic lymphadenectomy (39). In addition, some patients should also undergo para-aortic lymphadenectomy. Radiotherapy and hysterectomy are the recommended treatment options for patients with CC with LNM. Lin et al. (40) found that the 5-year overall survival and cancer-specific survival of patients who underwent hysterectomy were 57.8% and 50.0%, compared with 29.6% and 27.9% in the non-surgical group. Huang et al. (41) suggested that administering local radiotherapy to patients with distant metastasis might contribute to a better prognosis. Our study confirmed the benefits of surgery, lymphadenectomy, and chemoradiotherapy in patients with CC with LNM.

This study had some limitations. First, prognostic factors such as complications, detailed treatment plan, treatment sequence, treatment duration, and recurrence score were not recorded by SEER. Second, due to the fact that we failed to collect relevant evidence, depth of stromal invasion and LVSI are known prognostic markers which are not reported in the present study. Then, the results of regression analysis may have inherent bias and error. Finally, the datasets we used to build and validate nomograms were from the same SEER database, and requires validation using a larger, multi-center sample. These limitations may greatly affect the applicability of this study to actual cases. Therefore, the results of our analysis should be interpreted with caution.

In conclusion, our study screened out risk factors associated with LNM and prognosis of patients with CC. The nomogram we constructed provided a quantitative and visual method to predict the individual risk and survival of patients with CC with LNM.

## Data availability statement

Publicly available datasets were analyzed in this study. This data can be found here: https://seer.cancer.gov/.

## Author contributions

MW designed the study and participated in the statistical analysis. MM performed data processing and statistical analysis. The article was written by LY. CL participated in generation of the manuscript and is the corresponding author. All authors contributed to the article and approved the submitted version.

## Acknowledgments

We thank all the staff who work with the SEER database at the National Cancer Institute. We thank the Charlesworth team for polishing and editing our manuscript. (http://www.cwauthors.com/frontiers/)

## Conflict of interest

The authors declare that the research was conducted in the absence of any commercial or financial relationships that could be construed as a potential conflict of interest.

## Publisher’s note

All claims expressed in this article are solely those of the authors and do not necessarily represent those of their affiliated organizations, or those of the publisher, the editors and the reviewers. Any product that may be evaluated in this article, or claim that may be made by its manufacturer, is not guaranteed or endorsed by the publisher.
